# INTRODUCING NURSES IN EXPANDED ROLES IN LONG-TERM CARE FACILITIES

**DOI:** 10.1093/geroni/igae098.1953

**Published:** 2024-12-31

**Authors:** Franziska Zúñiga, Kornelia Kotkowski, Raphaelle-Ashley Guerbaai, Thekla Brunkert

**Affiliations:** University of Basel, Basel, Basel-Stadt, Switzerland; University of Basel, Basel, Basel-Stadt, Switzerland; Monash University, Frankston, Victoria, Australia; University of Lucerne, Luzern, Luzern, Switzerland

## Abstract

INTERCARE is a nurse-led care model introducing registered nurses in expanded roles (nurses with advanced education and training beyond the traditional scope of practice for registered nurses) into long-term care facilities (LTCFs) to strengthen geriatric expertise and reduce unplanned hospitalizations. INTERCARE nurses promote the core components of INTERCARE, such as interprofessional collaboration. INTERCARE was implemented in 11 LTCFs in Switzerland from 2018 to 2020 and tested in an effectiveness-implementation hybrid-type II design. Six months post-implementation, care staff (n=108) were interviewed about their perception of the INTERCARE nurse’s role. INTERCARE nurses (n=18) were interviewed at the end of the study to explore the content of their work and the factors supporting their role adoption. Staff reported high acceptability of INTERCARE nurses, feeling supported in daily practice and experiencing increased confidence in skills, such as responding to acute situations. INTERCARE nurses provided regular coaching to care teams in complex resident situations and guided interprofessional collaboration by supporting phone calls with physicians. They considered the ongoing training during the study as important to expand their clinical and leadership skills. Bi-weekly one-to-one coaching sessions, where they practiced analyzing and solving problems and additional exchanges with peers, were crucial in sustaining them in their challenging role. In conclusion, nurses in expanded roles provide a supportive environment for care staff to enhance their skills and provide quality care. Peer support and coaching are pivotal in sustaining these expanded roles in the demanding low-resource LTC environment.

